# The effects of agomelatine and imipramine on liver cytochrome P450 during chronic mild stress (CMS) in the rat

**DOI:** 10.1007/s43440-020-00151-w

**Published:** 2020-08-03

**Authors:** Anna Haduch, Ewa Bromek, Marta Rysz, Renata Pukło, Mariusz Papp, Piotr Gruca, Magdalena Łasoń, Monika Niemczyk, Władysława A. Daniel

**Affiliations:** 1 Department of Pharmacokinetics and Drug Metabolism, Maj Institute of Pharmacology, Polish Academy of Sciences, Smętna 12, 31-343 Kraków, Poland; 2 Department of Pharmacology, Maj Institute of Pharmacology, Polish Academy of Sciences, Smętna 12, 31-343 Kraków, Poland

**Keywords:** Chronic mild stress (CMS), Agomelatine, Imipramine, Liver, Cytochrome P450 expression, Cytochrome P450 activity

## Abstract

**Background:**

The aim of our research was to determine the effects of chronic treatment with the atypical antidepressant agomelatine on the expression and activity of liver cytochrome P450 (CYP) in the chronic mild stress (CMS) model of depression, and to compare the results with those obtained for the first-generation antidepressant imipramine.

**Methods:**

Male Wistar rats were subjected to CMS for 7 weeks. Imipramine (10 mg/kg ip/day) or agomelatine (40 mg/kg ip/day) was administered to nonstressed or stressed animals for 5 weeks (weeks 3–7 of CMS). The levels of cytochrome P450 mRNA, protein and activity were measured in the liver.

**Results:**

Agomelatine and imipramine produced different broad-spectrum effects on cytochrome P450. Like imipramine, agomelatine increased the expression/activity of CYP2B and CYP2C6, and decreased the CYP2D activity. Unlike imipramine, agomelatine raised the expression/activity of CYP1A, CYP2A and reduced that of CYP2C11 and CYP3A. CMS modified the effects of antidepressants at transcriptional/posttranscriptional level; however, the enzyme activity in stressed rats remained similar to that in nonstressed animals. CMS alone decreased the CYP2B1 mRNA level and increased that of CYP2C11.

**Conclusion:**

We conclude the following: (1) the effects of agomelatine and imipramine on cytochrome P450 are different and involve both central and peripheral regulatory mechanisms, which implicates the possibility of drug–drug interactions; (2) CMS influences the effects of antidepressants on cytochrome P450 expression, but does not change appreciably their effects on the enzyme activity. This suggests that the rate of antidepressant drug metabolism under CMS is similar to that under normal conditions.

## Introduction

Cytochrome P450 (CYP) enzymes play an important role in the oxidative metabolism of endogenous substances (e.g. steroids, arachidonic acid, vitamins) and exogenous compounds including drugs. The physiological expression of liver cytochrome P450 is regulated via the hormonal and immune systems [1]. Our earlier studies showed an important role of the brain dopaminergic [2], noradrenergic [3, 4] and serotonergic [5–9] systems in the physiological neuroendocrine regulation of cytochrome P450 expression in the liver. Those central mechanisms may also be involved in the enzyme regulation by psychotropic drugs or stress.

Stress produces changes in the functioning of the nervous, endocrine and immune systems, which may differ depending on its nature, intensity and duration. Stress engages the brain catecholaminergic and other neurotransmitter systems and, in consequence, it affects the endocrine pathways (e.g., stimulation of the HPA axis) and immune responses (i.e. increased production of proinflammatory cytokines) [10–13]. Stress-evoked changes in these systems may affect cytochrome P450 expression and activity and modify the effect of psychotropic drugs on the enzyme. Our recent studies have indicated that chronic mild stress (CMS) affects liver cytochrome P450 and modifies the action of the atypical neuroleptic lurasidone or the antidepressants venlafaxine and escitalopram, on cytochrome P450 in the liver or brain [14, 15].

It seems, therefore, interesting to find out the effects of chronic treatment with the novel antidepressant and antianxiety drug agomelatine [16–19] on liver cytochrome P450 at normal conditions and under CMS, with reference to the model antidepressant imipramine.

Imipramine, a dibenzoazepine derivative, is a noradrenaline and serotonin reuptake inhibitor having also some antagonistic properties at noradrenergic α_1_, histaminergic H_1_ and muscarinic acetylcholine M_1_ receptors [20]. Agomelatine, a naphthalene analogue of melatonin, is known as an antagonist of serotonergic 5-HT_2C_ receptors and agonist of melatonergic MT_1_ and MT_2_ receptors, and these two antagonistic/agonistic receptor effects give a synergistic beneficial therapeutic outcome [21–23]. By blocking 5-HT_2C_ receptors located on GABA-ergic interneurons in the brain stem, agomelatine disinhibits dopamine and noradrenaline release in the prefrontal cortex [24], while by stimulating melatonergic receptors in the suprachiasmatic nucleus agomelatine exerts its chronobiotic effect (“resynchronizing” circadian rhythms) [25, 26]. Chronic treatment with agomelatine increases neural plasticity and adult neurogenesis, in particular in the hippocampus [27, 28]. By acting on those serotonergic and melatonergic receptors the antidepressant increases the contents of brain-derived neurotrophic factor [29, 30] and reduces release of glutamate in limbic structures [31, 32]. The synergy of 5-HT_2C_ receptor antagonism and melatonergic receptor agonism is advantageous for the abovementioned pharmacological effects of agomelatine [33–35], as well as for the treatment of depression and anxiety.

Imipramine is known to be metabolized by a few CYP enzymes, i.e. by CYP2D6-catalyzed aromatic hydroxylation as well as CYP1A2-, CYP2C19- and CYP3A4-mediated *N*-demethylation [36, 37]. At pharmacological concentrations, it affects cytochrome P450 enzymes in the liver via different mechanisms [38–43]. However, the effect of long-term treatment with this drug (5 weeks, mimicking clinical conditions) on cytochrome P450 activity at normal conditions and under stress has not been studied so far.

Interactions between agomelatine and cytochrome P450 are less known. The drug is mainly metabolized by CYP1A2 and to a much lesser extent by CYP2C9 and CYP2C19 via hydroxylation and demethylation to form inactive metabolites. Moreover, CYP1A2 and CYP3A4 contribute to the formation of GSH adducts and hydrazones [44, 45]. Potential effects of agomelatine on cytochrome P450 at pharmacological/therapeutic concentrations are not known; however, it has been mentioned that high oral doses (≥ 125 mg/kg) induce cytochrome P450 in rodents and monkeys [46]. Moreover, clinical trials indicate that the risk of drug-induced hepatotoxicity is greater for agomelatine than for other antidepressants [47, 48], which may be connected with epoxide formation [44]. Both imipramine and agomelatine show antidepressant- and anxiolytic-like activity in animal models of depression or anxiety [49–52]. The aim of our research was to determine the effects of chronic treatment with agomelatine on the expression and activity of liver cytochrome P450 in the CMS model of depression [53] and to compare the results with those obtained for imipramine.

## Materials and methods

### Animals

All procedures used in this study were in accordance with the 86/609 EEC Directive, and were approved by the Local Bioethics Commission at the Maj Institute of Pharmacology, Polish Academy of Sciences. The experiments were carried out on male Wistar Han rats (Charles River Laboratories, Sulzfeld, Germany), weighing 280–300 g. The animals were singly housed with food and water freely available, and were maintained on a 12-h light/dark cycle (light on at 08.00 h) under conditions of constant temperature (22 ± 2 °C) and humidity (50 ± 5%).

### Drugs and chemicals

Imipramine (hydrochloride) was provided by Sigma (St. Louis, MO, USA), agomelatine by Carbosynth (Berkshire, UK). NADP, NADPH, glucose-6-phosphate-dehydrogenase, glucose-6-phosphate, caffeine and its metabolites, bufuralol and its metabolite 1′-hydroxybufuralol and RNA-free water were purchased from Sigma (St. Louis, MO, USA). Testosterone and its metabolites were provided by Steraloids (Newport, KY, USA). Warfarin was donated by Merck (Darmstadt, Germany), while 7-hydroxywarfarin was synthesized at our Institute [24]. The primary mouse monoclonal anti-rat CYP2C6, the rabbit polyclonal anti-rat CYP2C11 and anti-human CYP2D6 antibodies were obtained from Abcam (Cambrige, UK), the rabbit anti-rat CYP3A1 and CYP3A2 antibodies came from Millipore (Temecula, USA). The polyclonal primary rabbit anti-human CYP2A13, the monoclonal mouse anti-rat CYP2B1/2B2 and the polyclonal anti-rat β-actin antibodies were from Santa Cruz Biotechnology (Dallas, TX, USA). The polyclonal primary anti-rat CYP1A1 antibody was from Daiichi Pure Chemicals (Tokyo, Japan). Horseradish peroxidase-labeled secondary antibodies and goat anti-mouse were from Jackson ImmunoResearch (West Grove, PA, USA) and goat anti-rabbit from Vector Laboratories (Burlingame, CA, USA). Rat cDNA-expressed CYP1A1, CYP2B1, CYP2C6, CYP2C11, CYP3A1/23, CYP3A2, human cDNA-expressed CYP2D6 (Supersomes) and pooled human liver microsomes were from Gentest Corp. (Woburn, MA, USA). The chemiluminescence reagents LumiGlo kit came from KPL (Gaithersburg, MD, USA). A mirVana kit, TaqMan assays and the TaqMan Gene Expression Master Mix from Life Technologies (Carlsbad, CA, USA), and a Transcriptor High-Fidelity cDNA synthesis kit from Roche Diagnostics (Indianapolis, IN, USA) were used for RNA isolation and mRNA estimation.

### In vivo experiment and preparation of liver microsomes

CMS experiments and sucrose consumption test were performed according to the methods described previously [15, 54]. Briefly, rats were subjected to the CMS procedure for 7 weeks. The weekly stress procedure included two periods of food or water deprivation, 45 degree cage tilt, intermittent illumination, soiled cage (water in sawdust bedding), one period of paired housing, two periods of low-intensity stroboscopic illumination, and three periods of no stress, according to the CMS protocol [54, 55]. All stressors lasted for 10–14 h and were applied individually and incessantly, day and night. After 2 weeks of initial stress, both nonstressed and stressed animals were divided into three subgroups and for the next 5 weeks received once-daily intraperitoneal injections (ip) of vehicle (saline, 1 ml/kg), imipramine (10 mg/kg) or agomelatine (40 mg/kg). The applied doses were optimal to achieve an antidepressant effect in the rat CMS model, as measured by sucrose consumption test [49, 52]. Stress was continued throughout the time of the treatment (5 weeks). After the stress procedure and antidepressant treatment were completed, animals underwent biochemical studies into cytochrome P450 expression and activity in the liver. All animals were killed by decapitation (24 h after the last dose). Their livers were quickly isolated, frozen in dry ice and stored at -80 °C. Liver microsomes were prepared from individual rats by differential centrifugation (11,000×*g* and 2 × 100,000×*g*) in a 20 mM Tris/KCl buffer (pH 7.4), including washing with 0.15 M KCl, as described previously [38]. The above procedure deprives microsomes of in vivo administered drugs.

### Determination of CYP enzyme activity in the liver

The activities of CYP enzymes were studied at the linear conditions as concerns time, protein and substrate concentration, according to the previously optimized conditions. Incubations were carried out in a medium containing liver microsomes (ca. 1 mg of protein/ml) and NADP or NADPH-generating system as described previously. The activity of CYP1A was determined by measuring the rate of caffeine metabolism (C-8-hydroxylation and 3-*N*-demethylation) at a substrate concentration of 100 μM and incubation time of 50 min. Caffeine and its metabolites were analyzed by HPLC with UV detection [56]. The activity of CYP2C6 was studied by measuring the rate of warfarin 7-hydroxylation at a substrate concentration of 60 μM and incubation time of 15 min. Warfarin and its metabolite were analyzed by HPLC with fluorescence detection [40]. The activity of CYP2D was estimated by measuring the rate of bufuralol 1′-hydroxylation at a substrate concentration of 10 μM and incubation time of 10 min. Bufuralol and its metabolite were analyzed by HPLC with fluorescence detection [57, 58]. The activities of CYP2A, CYP2B, CYP2C11 and CYP3A were studied by measuring the rate of cytochrome P450 enzyme-specific reactions: the 7α-, 16β-, 2α- and 16α-, and 6β-hydroxylation of testosterone, respectively, at a substrate concentration of 100 μM and incubation time of 15 min, as described previously. Testosterone and its metabolites were analyzed by HPLC with UV detection [41–43].

### Analysis of CYP proteins in the liver

The protein levels of CYP1A, CYP2A, CYP2C11, CYP2B, CYP2D, CYP3A1/23 and CYP3A2 in the liver microsomes (10 µg of proteins) of control and antidepressant-treated rats were estimated by Western immunoblot analysis, as previously described [5, 6, 14]. Monoclonal anti-rat CYP2B and CYP2C6, polyclonal anti-rat CYP1A1, CYP2C11, CYP3A1/23 or CYP3A2 antibodies, anti-human CYP2A13 (reacting with rat CYP2A) and CYP2D6 (reacting with rat CYP2D) antibodies, and a secondary antibody (a species-specific horseradish peroxidase-conjugated anti-IgG) were used. Rat cDNA-expressed CYP1A1, CYP2B1, CYP2C6, CYP2C11 (5 µg), CYP3A1/23, CYP3A2 (1 µg) and human cDNA-expressed CYP2D6 (1 µg) or pooled human liver microsomes (20 µg, reacting with anti-human CYP2A13 antibodies) were used as respective standards. The intensity of the bands on a nitrocellulose membrane was quantified with the Luminescent Image Analyzer and the data were normalized to protein loading based on the β-actin levels [5, 6].

### Isolation of liver RNA and quantitative real-time polymerase chain reaction (qRT-PCR) measurements

RNA was isolated from the liver, and quantitative real-time PCR was performed as described previously [6]. Briefly, frozen liver tissue was homogenized, and total RNA was extracted using a mirVana isolation kit and then the first-strand cDNA products were generated using a Transcriptor High Fidelity cDNA Synthesis Kit. The expression of the genes encoding the cytochrome P450 enzymes (*CYP1A1, CYP1A2, CYP2A1, CYP2A2, CYP2B1, CYP2B2, CYP2C11, CYP2C6, CYP3A1/23, CYP3A2, CYP2D1* and *CYP2D2*) and the reference genes *glyceraldehyde-3-phosphate dehydrogenase *(*GAPDH*) and *β-actin* were detected by real-time PCR using commercially available TaqMan Gene Expression Master Mix and species-specific TaqMan type probes and primers. The gene names for the tested *CYP* genes and two reference genes with identification numbers of the TaqMan primers used in the study are as follows: *CYP1A1* (Rn01418021_g1), *CYP1A2* (Rn00561082_m1), *CYP2A1* (Rn04219367_m1), *CYP2A2* (Rn00562207_m1), *CYP2C6* (Rn03417171_gH), *CYP2B1* (Rn01457880_m1), *CYP2B2* (Rn02786833_m1), *CYP2C11* (Rn01502203_m1), *CYP3A1/23* (Rn03062228_m1), *CYP3A2* (Rn00756461_m1), *CYP2D1* (Rn01775090_mH), *CYP2D2* (Rn00562419_m1), *GAPDH* (Rn01462662_g1) and *β-actin* (Rn00667869_m1). Real-time PCR runs were performed using the Bio-Rad CFX96 PCR system (Bio-Rad, Hercules, CA, USA) as described previously [6]. The level of the *CYP* transcripts was normalized to the *β-actin* expression (*β-actin* was chosen as a reference gene since its expression was stable compared to that of *GAPDH* after the applied treatments), and the relative quantification was obtained by a comparative delta–delta *C*_t_ method (${2}^{{ - \Delta \Delta C_{{\text{t}}} }}$).

### Statistical analysis of data

All data are reported as the means (± S.E.M.). The results of CYP protein levels were analyzed using one-way analysis of variance (ANOVA) followed by the post hoc Duncan’s test. Because of limited area on the electrophoresis gel or nitrocellulose membrane, separate Western blot analyses of CYP proteins for non-stressed and stressed animals were carried out. Thus, the CYP protein levels were tested separately for nonstressed (nonstressed control, imipramine and agomelatine) and stressed (stressed control, imipramine and agomelatine) groups of animals. Statistically significant values of *F* and *p* generated in the one-way ANOVA analysis are shown in Table 2. But the results of *CYP* mRNA levels and activity were analyzed using two-way ANOVA followed by the post hoc Duncan’s test, since in this case nonstressed and stressed groups were tested jointly. Therefore, two factors were taken into account in the two-way ANOVA: factor I (stressed/nonstressed) and factor II (imipramine/agomelatine/saline control). Statistically significant values of *F* and *p* derived from the two-way ANOVA analysis of *CYP* mRNA levels and activity are shown in Tables 1 and 3, respectively. All the *p* values of Duncan’s test are presented in the figure captions. The results were considered as statistically significant when *p* < 0.05.

**Table 1:** Statistically significant values of *F* and *p* derived from the two-way analysis of variance (ANOVA) carried out to analyze the results of *CYP* mRNA levels for: factor I—–stressed/nonstressed, factor II—IMI/AGM/saline control, and for the interaction between factors I and II–stressed/nonstressed × IMI/AGM/saline control

CYP	Stressed/nonstressed		IMI/AGM/saline		Stressed/nonstressed × IMI/AGM/saline	
	*F*	*p*	*F*	*p*	*F*	*p*
1A1	*F*_1,36_ = 0.081	0.777478	*F*_2,36_ = 1.712	0.194960	***F***_**2,36**_** = 4.855**	**0.013590**
1A2	*F*_1,35_ = 36.8005	0.070001	***F***_**2,35**_** = 17.1684**	**0.000006**	***F***_**2,35**_** = 14.4333**	**0.0000027**
2A1	*F*_1,38_ = 0.58164	0.450380	***F***_**2,38**_** = 9.42655**	**0.000474**	*F*_2,38_ = 0.89181	0.418319
2A2	*F*_1,38_ = 2.9081	0.096300	***F***_**2,38**_** = 5.4948**	**0.008015**	*F*_2,38_ = 3.3756	0.044730
2B1	***F***_**1,34**_** = 19.044**	**0.000113**	***F***_**2,34**_** = 46.665**	**0.000000**	*F*_2,34_ = 1.017	0.372560
2B2	*F*_1,30_ = 3.8644	0.059000	***F***_**2,30**_** = 22.6668**	**0.000001**	*F*_2,30_ = 0.2539	0.777443
2C6	*F*_1,30_ = 3.232177	0.082277	***F***_**2,30**_** = 4.060722**	**0.027497**	***F***_**2,30**_** = 5.404912**	**0.009893**
2C11	***F***_**1,34**_** = 3.81696**	**0.049003**	***F***_**2,34**_** = 14.82431**	**0.000023**	***F***_**2,34**_** = 4.35875**	**0.020646**
2D1	*F*_1,36_ = 0.620	0.436594	*F*_2,36_ = 8.233	0.125700	*F*_2,36_ = 0.759	0.476247
2D2	***F***_**1,36**_** = 5.7272**	**0.022043**	***F***_**2,36**_** = 3.4908**	**0.041151**	*F*_2,36_ = 2.8598	0.070361
3A1	***F***_**1,36**_** = 5.2605**	**0.027759**	***F***_**2,36**_** = 6.3422**	**0.0043681**	*F*_2,36_ = 2.1900	0.126600
3A2	*F*_1,32_ = 3.42780	0.073355	***F***_**2,32**_** = 26.968**	**0.000000**	*F*_2,32_ = 0.52249	0.598017

The values of *F* and *p* marked in bold indicate statistically significant changes in the mRNA levels of *CYPs* within and between studied factors

*IMI* imipramine, *AGM* agomelatine

**Table 2:** Statistically significant values of *F* and *p* derived from the one-way ANOVA carried out to analyze the results of CYP protein levels

CYP	Nonstressed Saline/IMI/AGM		Stressed Saline/IMI/AGM	
	*F*	*p*	*F*	*p*
1A	***F***_**2,21**_** = 14.092**	**0.0001**	***F***_**2,21**_** = 26.075**	**0.0000**
2A	***F***_**2,15**_** = 16.442**	**0.0002**	*F*_2,15_ = 1.677	0.2202
2B	***F***_**2,15**_** = 17.713**	**0.0001**	***F***_**2,15**_** = 60.471**	**0.0000**
2C6	***F***_**2,15**_** = 48.480**	**0.0000**	***F***_**2,15**_** = 50.671**	**0.0000**
2C11	***F***_**2,15**_** = 63.026**	**0.0000**	***F***_**2,15**_** = 38.863**	**0.0000**
2D	*F*_2,15_ = 0.623	0.5503	*F*_2,15_ = 3.938	0.0622
3A1	***F***_**2,21**_** = 42.661**	**0.0000**	***F***_**2,20**_** = 18.703**	**0.0000**
3A2	***F***_**2,15**_** = 35.051**	**0.0000**	***F***_**2,15**_** = 55.456**	**0.0000**

The CYP protein levels were tested separately for nonstressed (saline control, imipramine and agomelatine) and stressed (saline control, imipramine and agomelatine) groups of animals. The values of *F* and *p* marked in bold indicate statistically significant changes in the CYP protein level within studied factor

**Table 3:** Statistically significant values of *F* and *p* derived from the two-way analysis of variance (ANOVA) carried out to analyze the results of CYP activity levels for: factor I—stressed/nonstressed, factor II—IMI/AGM/saline control, and for the interaction between factors I and II—stressed/nonstressed × IMI/AGM/saline control

CYP	Stressed/nonstressed		IMI/AGM/saline		Stressed/nonstressed × IMI/AGM/saline	
	*F*	*p*	*F*	*p*	*F*	*p*
1A	*F*_1,54_ = 0.1953 F_1,54_ = 0.1868	0.660323 0.667319	***F***_**2,54**_** = 9.8423** ***F***_**2,54**_** = 4.2945**	**0.000227** **0.018585**	*F*_2,54_ = 0.0487 *F*_2,54_ = 0.4035	0.952490 0.669987
2A	*F*_1,53_ = 0.602	0.441280	***F***_**2,53**_** = 27.298**	**0.000000**	*F*_2,53_ = 1.459	0.241631
2B	*F*_1,53_ = 0.7534	0.389308	***F***_**2,53**_** = 7.4751**	**0.001381**	*F*_2,53_ = 0.0056	0.994380
2C6	*F*_1,52_ = 1.933	0.170303	***F***_**2,52**_** = 14.051**	**0.000013**	*F*_2,52_ = 1.568	0.218204
2C11	***F***_**1,50**_** = 4.6492** *F*_1,45_ = 3.0059	**0.035904** 0.089809	***F***_**2,50**_** = 24.3904** ***F***_**2,45**_** = 20.6261**	**0.000000** **0.000000**	*F*_2,50_ = 0.1450 *F*_2,45_ = 0.1266	0.865420 0.881420
2D	*F*_1,52_ = 0.9777	0.327337	***F***_**2,52**_** = 65.0573**	**0.000000**	*F*_2,52_ = 1.2554	0.293456
3A	*F*_1,48_ = 2.825	0.099311	***F***_**2,48**_** = 10.129**	**0.000214**	*F*_2,48_ = 0.954	0.392213

The values of *F* and *p* marked in bold indicate statistically significant changes in the activity of CYPs within and between studied factors

*IMI* imipramine, *AGM* agomelatine

## Results

### The effect of imipramine and agomelatine on the CYP1A expression and activity in the liver of nonstressed and stressed rats

**Fig. 1:**
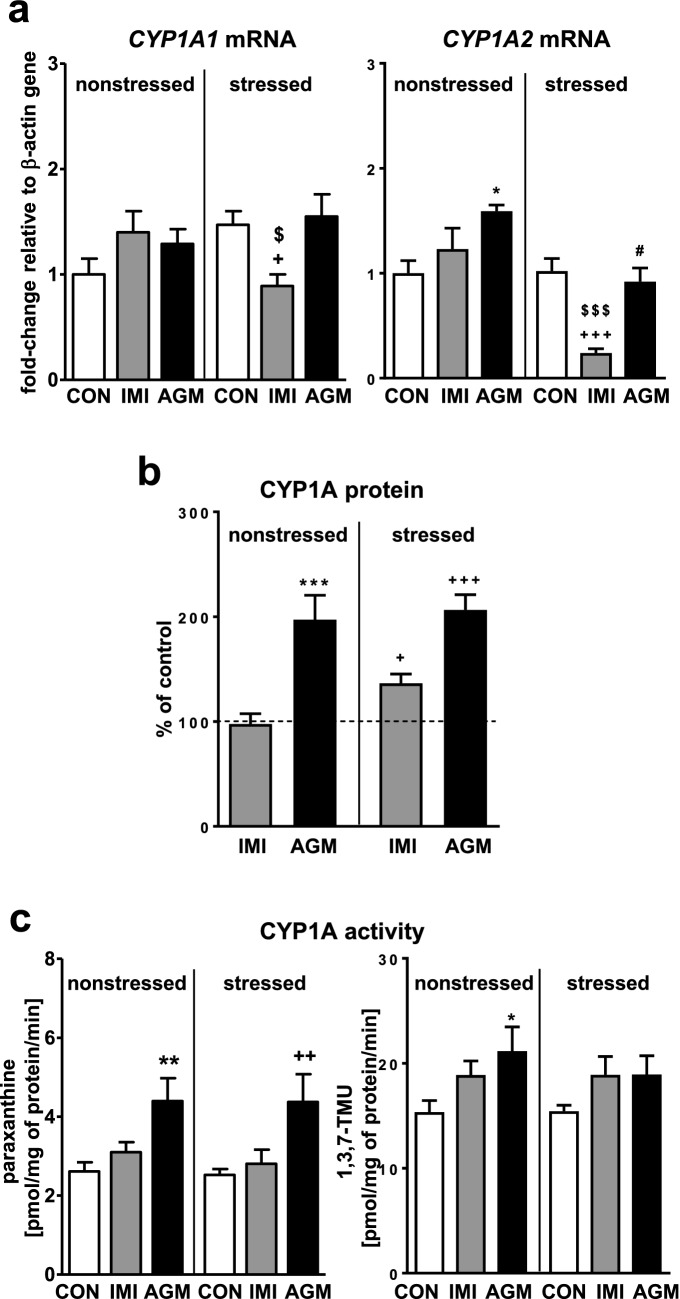
The influence of a 5-week treatment with imipramine or agomelatine on the expression and activity of CYP1A in the liver from the chronic mild stress (CMS) model. **a** The mRNA expression level of *CYP1A1* and *CYP1A2.* Results are expressed as the fold-change in relation to the housekeeping gene *β-actin* (*n* = 6–8), the outcome of two-way ANOVA is shown in Table 1; **b** CYP1A protein level (*n* = 8), the outcome of one-way ANOVA is shown in Table 2; **c** CYP1A activity measured as a rate of caffeine 3-*N*-demethylation (n = 9–10), the outcome of two-way ANOVA is shown in Table 3. Results are shown as the means ± S.E.M. Duncan’s test: statistical significance is shown as *p < 0.05, **p < 0.01, ***p < 0.001 compared to the nonstressed control; ^+^*p* < 0.05, ^++^*p* < 0.01, ^+++^*p* < 0.001 compared to the stressed control; ^$^*p* < 0.05, ^$$$^*p* < 0.001 compared to the nonstressed imipramine-treated rats; ^#^*p* < 0.01 compared to the nonstressed agomelatine-treated rats. The representative CYP1A protein bands of the Western immunoblot analysis are shown in Fig. 8. *CON* control, *IMI* imipramine, *AGM* agomelatine, *1,3,7-TMU* 1,3,7-trimethyluric acid

Chronic mild stress (CMS) did not affect the expression or activity of CYP1A enzymes (Fig. 1a, c, respectively). Imipramine tended to elevate the mRNA levels of both *CYP1A1* and *CYP1A2* in nonstressed rats, but decreased those levels in stressed animals (Fig. 1a). Thus the expression of *CYP1A1/2* genes under antidepressant treatment was significantly lower in stressed animals whereas the protein level of CYP1A gently increased in stressed animals only (Fig. 1b). The activity of CYP1A (measured as the caffeine C-8-hydroxylation and 3-*N*-demethylation) remained unchanged after imipramine treatment (Fig. 1c), though a tendency to increase was observed in both groups of rats (nonstressed and stressed).

Agomelatine significantly increased the mRNA level of *CYP1A2* in nonstressed rats (Fig. 1a), not affecting significantly *CYP1A1* mRNA. The *CYP1A1/2* mRNA levels remained unchanged in stressed animals under antidepressant treatment, but the *CYP1A2* mRNA level was lower compared to nonstressed animals treated with agomelatine. However, the antidepressant increased the CYP1A protein level (Fig. 1b) and activity (Fig. 1c) in both groups of rats.

### The effect of antidepressants on the CYP2A expression and activity in the liver of nonstressed and stressed rats

**Fig. 2:**
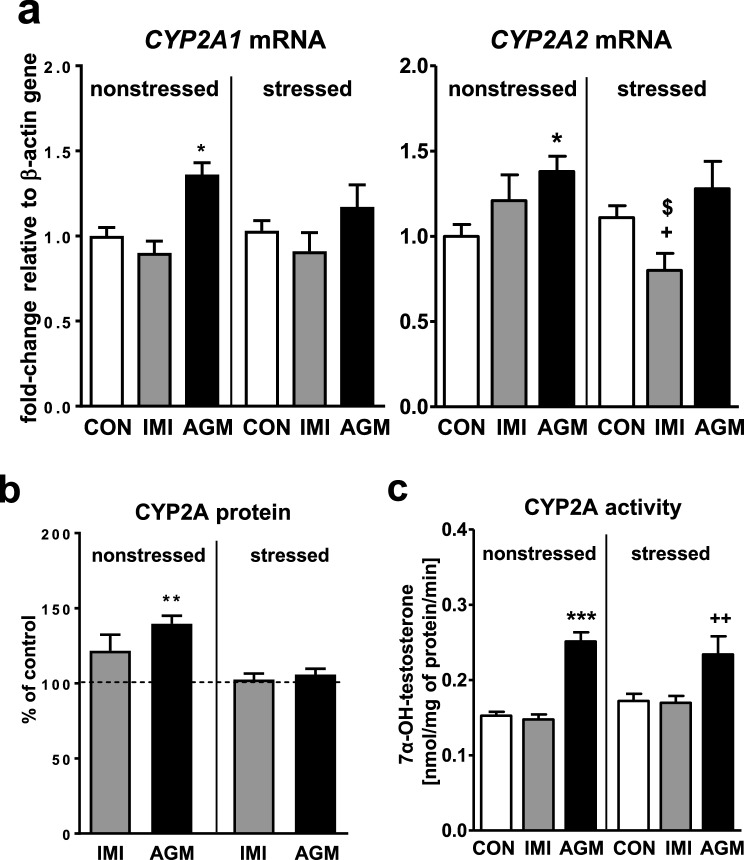
The influence of a 5-week treatment with imipramine or agomelatine on the expression and activity of CYP2A in the liver from the chronic mild stress (CMS) model. **a** The mRNA expression level of *CYP2A1* and *CYP2A2.* Results are expressed as the fold-change in relation to the housekeeping gene *β-actin* (*n* = 7–8), the outcome of two-way ANOVA is shown in Table 1; **b** CYP2A protein level (*n* = 6), the outcome of one-way ANOVA is shown in Table 2; **c** CYP2A activity measured as a rate of testosterone 7α-hydroxylation (*n* = 9–10), the outcome of two-way ANOVA is shown in Table 3. Results are shown as the means ± S.E.M. Duncan’s test: statistical significance is shown as *p < 0.05, ***p < 0.001 compared to the nonstressed control; ^+^*p* < 0.05, ^++^*p* < 0.01 compared to the stressed control; ^$^*p* < 0.05 compared to the nonstressed imipramine-treated rats. The representative CYP2A protein bands of the Western immunoblot analysis are shown in Fig. 8. *CON* control, *IMI* imipramine, *AGM* agomelatine

CMS did not exert any effect on the expression or activity of CYP2A enzymes (Fig. 2a, c, respectively). Imipramine did not affect the mRNA level of *CYP2A1* or *CYP2A2* in nonstressed rats, though gently decreased that of *CYP2A2* in stressed animals (Fig. 2a). The CYP2A protein level and activity (testosterone 7α-hydroxylation) remained unchanged after imipramine treatment (Fig. 2b, c, respectively).

Agomelatine moderately raised the *CYP2A1* and *CYP2A2* mRNAs (Fig. 2a) and CYP2A protein level (Fig. 2b) only in nonstressed rats, but significantly increased the activity of CYP2A in both nonstressed and stressed animals (Fig. 2c).

### The effect of antidepressants on the CYP2B expression and activity in the liver of nonstressed and stressed rats

**Fig. 3:**
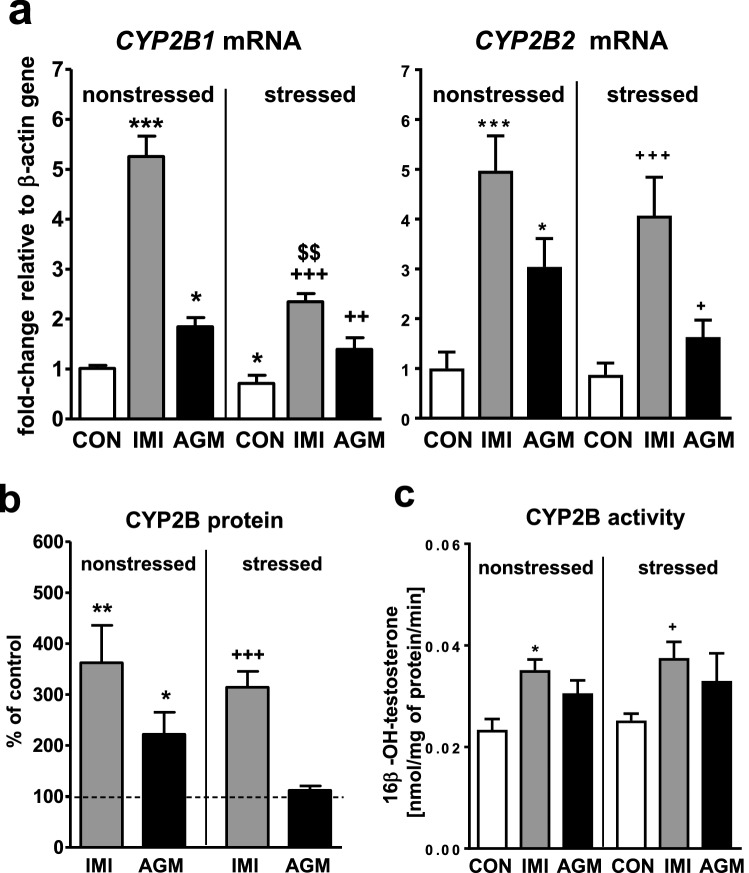
The influence of a 5-week treatment with imipramine or agomelatine on the expression and activity of CYP2B in the liver from the chronic mild stress (CMS) model. **a** The mRNA expression level of *CYP2B1* and *CYP2B2.* Results are expressed as the fold-change in relation to the housekeeping gene *β-actin* (*n* = 6–8), the outcome of two-way ANOVA is shown in Table 1; **b** CYP2B protein level (*n* = 6), the outcome of one-way ANOVA is shown in Table 2; **c** CYP2B activity measured as a rate of testosterone 16β-hydroxylation (*n* = 9–10), the outcome of two-way ANOVA is shown in Table 3. Results are shown as the means ± S.E.M. Duncan’s: statistical significance is shown as *p < 0.05, **p < 0.01, ***p < 0.001 compared to the nonstressed control; ^+^*p* < 0.05, ^++^*p* < 0.01, ^+++^*p* < 0.001 compared to the stressed control; ^$$^*p* < 0.01 compared to the nonstressed imipramine-treated rats. The representative CYP2B protein bands of the Western immunoblot analysis are shown in Fig. 8. *CON* control, *IMI* imipramine, *AGM* agomelatine

CMS decreased the *CYP2B1* mRNA level (Fig. 3a) not affecting the CYP2B activity (Fig. 3c). Imipramine strongly increased the *CYP2B1* and *CYP2B2* mRNA levels (up to fivefold) in nonstressed and stressed rats, and the effect on the *CYP2B1* mRNA level was more pronounced in nonstressed group (Fig. 3a). The antidepressant significantly increased the CYP2B protein level (Fig. 3b) and activity (Fig. 3c) measured as the testosterone 16β-hydroxylation rate in both nonstressed and stressed animals.

Agomelatine markedly increased the mRNA level of *CYP2B1* up to two-fold and *CYP2B2* up to three-fold in nonstressed and stressed rats (Fig. 3a). The CYP2B protein level was enhanced only in nonstressed animals (Fig. 3b). The CYP2B activity was not significantly changed by agomelatine treatment in both groups of rats, though a tendency to increase was observed (Fig. 3c).

### The effect of antidepressants on the CYP2C6 expression and activity in the liver of nonstressed and stressed rats

**Fig. 4:**
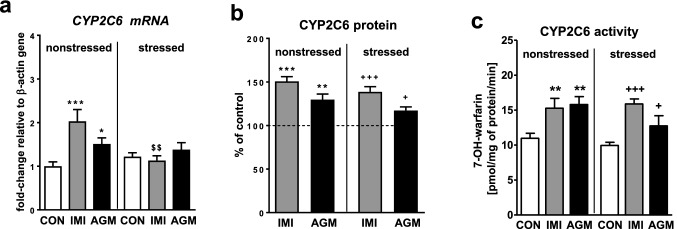
The influence of a 5-week treatment with imipramine or agomelatine on the expression and activity of CYP2C6 in the liver from the chronic mild stress (CMS) model. **a** The mRNA expression level of *CYP2C6.* Results are expressed as the fold-change in relation to the housekeeping gene *β-actin* (*n* = 6), the outcome of two-way ANOVA is shown in Table 1; **b** CYP2C6 protein level (*n* = 6), the outcome of one-way ANOVA is shown in Table 2; **c** CYP2C6 activity measured as a rate of warfarin 7-hydroxylation (n = 9–10), the outcome of two-way ANOVA is shown in Table 3. Results are shown as the means ± S.E.M. Duncan’s test: statistical significance is shown as *p < 0.05, **p < 0.01, ***p < 0.001 compared to the nonstressed control; ^+^*p* < 0.05, ^+ + +^*p* < 0.001 compared to the stressed control; ^$$^*p* < 0.01 compared to the nonstressed imipramine-treated rats. The representative CYP2C6 protein bands of the Western immunoblot analysis are shown in Fig. 8. *CON* control, *IMI* imipramine, *AGM* agomelatine

CMS did not influence the expression or activity of CYP2C6 (Fig. 4a, c, respectively). The CYP2C6 mRNA level was distinctly increased (up to twofold) by imipramine in nonstressed rats (Fig. 4a), whereas the antidepressant increased the protein level (Fig. 4b) and activity measured as the warfarin 7-hydroxylation rate (Fig. 4c) in both nonstressed and stressed animals.

Agomelatine moderately elevated the *CYP2C6* mRNA levels in nonstressed rats (Fig. 4a), but increased the CYP2C6 protein level (Fig. 4b) and activity (Fig. 4c) in both nonstressed and stressed groups of animals.

### The effect of antidepressants on the CYP2C11 expression and activity in the liver of nonstressed and stressed rats

**Fig. 5:**
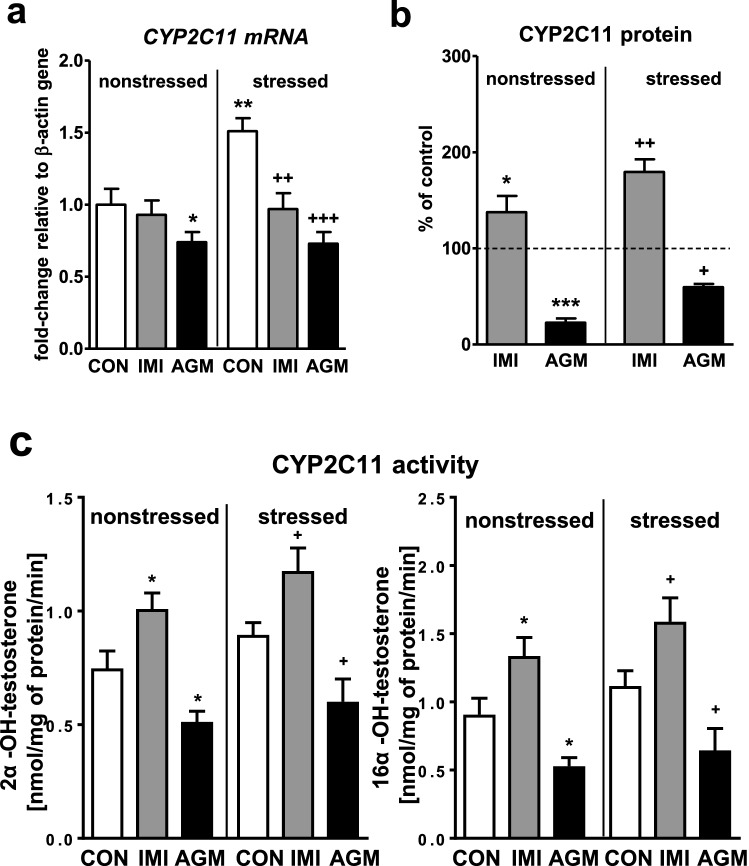
The influence of a 5-week treatment with imipramine or agomelatine on the expression and activity of CYP2C11 in the liver from the chronic mild stress (CMS) model. **a** The mRNA expression level of *CYP2C11.* Results are expressed as the fold-change in relation to the housekeeping gene *β-actin* (*n* = 6–8), the outcome of two-way ANOVA is shown in Table 1; **b** CYP2C11 protein level (*n* = 6), the outcome of one-way ANOVA is shown in Table 2; **c** CYP2C11 activity measured as a rate of testosterone 2α-hydroxylation (*n* = 8–10), the outcome of two-way ANOVA is shown in Table 3. Results are shown as the means ± S.E.M. Duncan’s test: statistical significance is shown as *p < 0.05, **p < 0.01, ***p < 0.001 compared to the nonstressed control; ^+^*p* < 0.05, ^++^*p* < 0.01, ^+++^*p* < 0.001 compared to the stressed control. The representative CYP2C11 protein bands of the Western immunoblot analysis are shown in Fig. 8. *CON* control, *IMI* imipramine, *AGM* agomelatine

CMS moderately increased the *CYP2C11* mRNA level in the rat liver (Fig. 5a) and showed such a tendency for the enzyme activity measured as the testosterone 2α- and 16α-hydroxylation rate (Fig. 5c). Imipramine treatment significantly decreased the *CYP2C11* mRNA level in stressed animals (Fig. 5a). At the same time the antidepressant increased the protein level (Fig. 5b) and activity (Fig. 5c) of CYP2C11 in both nonstressed and stressed groups of rats.

The *CYP2C11* mRNA was reduced a little by agomelatine in nonstressed rats, but distinctly dropped down in stressed animals (Fig. 5a). Accordingly, the enzyme protein (Fig. 5b) and activity (Fig. 5c) were also reduced by agomelatine in both treated groups.

### The effect of antidepressants on the CYP2D expression and activity in the liver of nonstressed and stressed rats

**Fig. 6:**
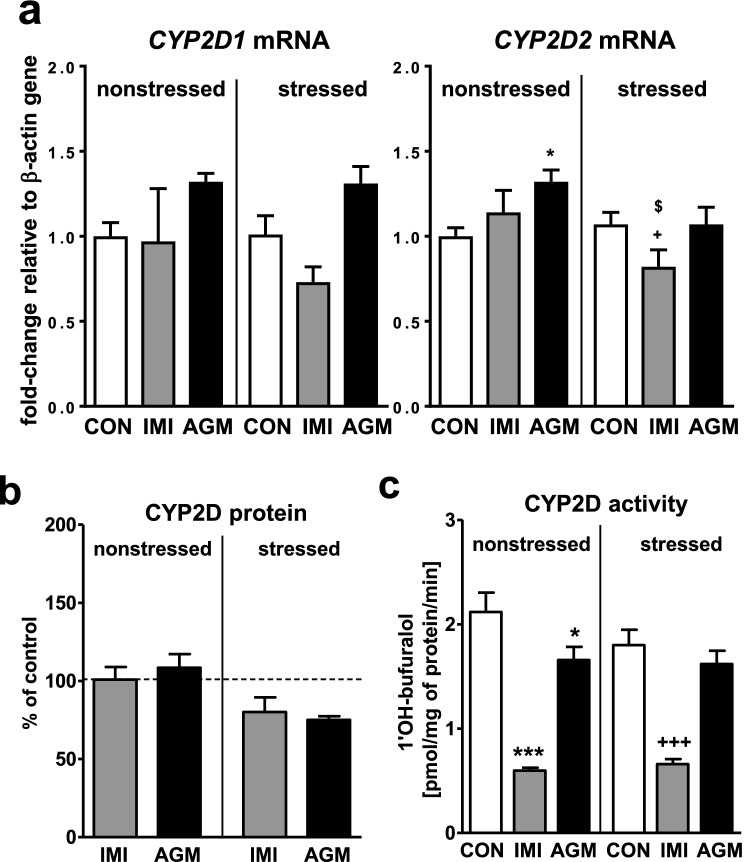
The influence of a 5-week treatment with imipramine or agomelatine on the expression and activity of CYP2D in the liver from the chronic mild stress (CMS) model. **a** The mRNA expression level of *CYP2D1* and *CYP2D2.* Results are expressed as the fold-change in relation to the housekeeping gene *β-actin* (*n* = 6–8), the outcome of two-way ANOVA is shown in Table 1; **b** CYP2D protein level (*n* = 6), the outcome of one-way ANOVA is shown in Table 2; **c** CYP2D activity measured as a rate of bufuralol 1′-hydroxylation (*n* = 9–10), the outcome of two-way ANOVA is shown in Table 3. Duncan’s test (**a, b, c**): statistical significance is shown as *p < 0.05, ***p < 0.001 compared to the nonstressed control; ^+^*p* < 0.05, ^+++^*p* < 0.001 compared to the stressed control; $ *p* < 0.05 compared to the nonstressed imipramine-treated rats. The representative CYP2D protein bands of the Western immunoblot analysis are shown in Fig. 8. *CON* control, *IMI* imipramine, *AGM* agomelatine

CMS had no effect on CYP2D1/2 expression or activity (Fig. 6a, c, respectively). The *CYP2D2* mRNA level was slightly decreased (Fig. 6a), while that of *CYP2D1* showed such a tendency in imipramine-treated rats under stress. The CYP2D protein level was not changed by imipramine (Fig. 6b). However, the activity of CYP2D (the rate of bufuralol 1′-hydroxylation) was decreased by the antidepressant in both groups of animals (Fig. 6c).

Agomelatine gently enhanced the mRNA level of *CYP2D2* in nonstressed animals only (Fig. 6a). The CYP2D protein level was not significantly changed (tended to decrease in stressed rats, Fig. 6b) and the enzyme activity was slightly diminished by agomelatine (Fig. 6c) in nonstressed animals only.

### The effect of antidepressants on the CYP3A expression and activity in the liver of nonstressed and stressed rats

**Fig. 7:**
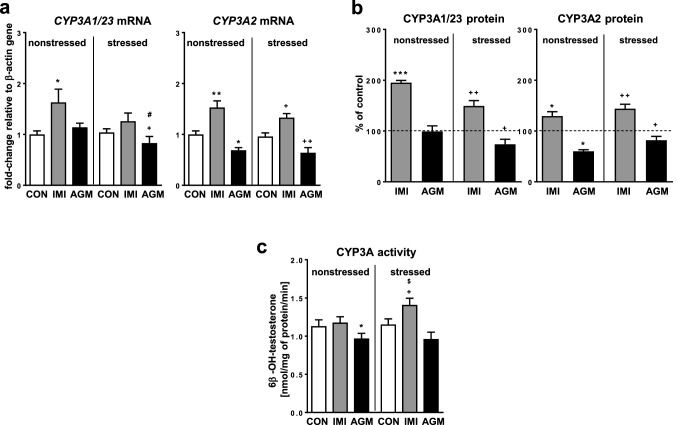
The influence of a 5-week treatment with imipramine or agomelatine on the expression and activity of CYP3A in the liver from the chronic mild stress (CMS) model. **a** The mRNA expression level of *CYP3A1/23* and *CYP3A2.* Results are expressed as the fold-change in relation to the housekeeping gene *β-actin* (*n* = 6–8), the outcome of two-way ANOVA is shown in Table 1; **b** CYP3A1/23 and CYP3A2 protein level (*n* = 6–8), the outcome of one-way ANOVA is shown in Table 2; **c** CYP3A activity measured as a rate of testosterone 6β-hydroxylation (*n* = 8–10), the outcome of two-way ANOVA is shown in Table 3. Duncan’s test: statistical significance is shown as *p < 0.05, **p < 0.01, ***p < 0.001 compared to the nonstressed control; ^+^*p* < 0.05, ^++^*p* < 0.01 compared to the stressed control; ^#^*p* < 0.05 compared to the nonstressed agomelatine-treated rats. The representative CYP3A1 and CYP3A2 protein bands of the Western immunoblot analysis are shown in Fig. 8. *CON* control, *IMI* imipramine, *AGM* agomelatine

CMS did not affect the mRNA level of *CYP3A1* and *CYP3A2* or the CYP3A activity, measured as the testosterone 6β-hydroxylation rate (Fig. 7a, c, respectively). Imipramine treatment distinctly increased the *CYP3A2* mRNA levels in nonstressed and stressed rats (Fig. 7a). The *CYP3A1/23* mRNA was significantly increased only in nonstressed group (Fig. 7a), and showed such a tendency in stressed group of animals. The antidepressant augmented the protein level of CYP3A1/23 and CYP3A2 in both nonstressed and stressed groups (Fig. 7b). Interestingly, the activity of CYP3A was not affected by imipramine in nonstressed rats, but it was enhanced in stressed animals (Fig. 7c).

Agomelatine significantly diminished the *CYP3A2* mRNA (Fig. 7a) and protein level (Fig. 7b) in both nonstressed and stressed rats. The antidepressant decreased the levels of *CYP3A1/23* mRNA (Fig. 7a) and protein (Fig. 7b) in stressed group only. Accordingly, agomelatine significantly decreased the CYP3A activity in nonstressed rats (Fig. 7c), and showed such a tendency in stressed animals.

**Fig. 8:**
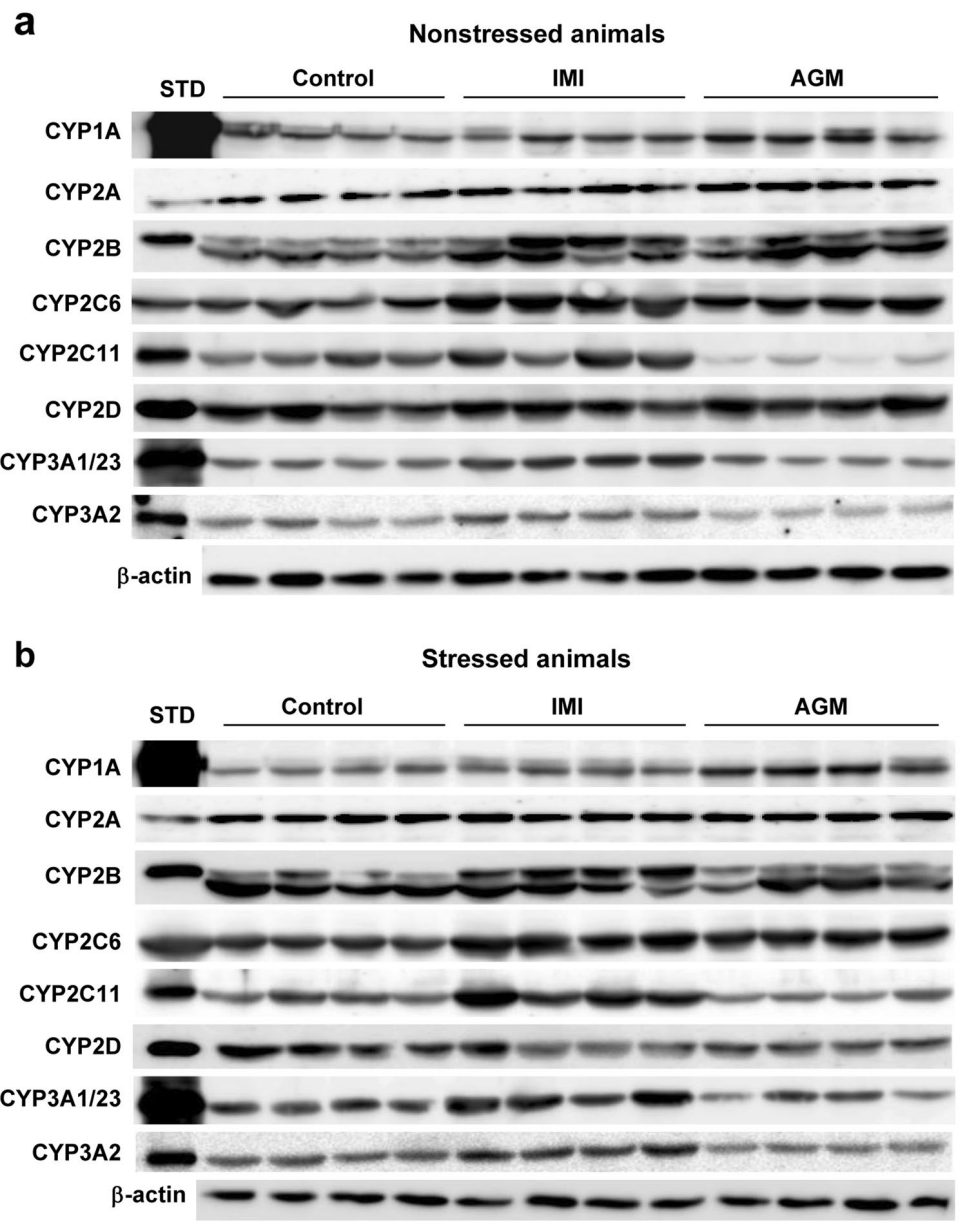
The effect of a 5-week treatment with imipramine or agomelatine on the intensity of protein bands of cytochrome P450 enzymes (CYP1A, CYP2A, CYP2B, CYP2C6 CYP2C11, CYP2D and CYP3A) in rat liver microsomes from nonstressed (**a**) and stressed (**b**) rats. Microsomal proteins were subjected to the Western immunoblot analysis. cDNA-expressed CYP enzymes were used as standards. The representative CYP protein bands in imipramine- or agomelatine-treated rats are shown. The presented results are typical of four separate rats per group. The mean values ± S.E.M. are shown in Figs. 1b, 2b, 3b, 4b, 5b, 6b and 7b. *IMI* imipramine, *AGM* agomelatine, *STD* standard

The representative CYP protein bands in imipramine- or agomelatine-treated rats, obtained as a result of Western blotting, are shown in Fig. 8a (nonstressed rats) and Fig. 8b (stressed rats).

Statistically significant values of *F* and *p* generated in the one-way ANOVA analysis of CYP protein levels are shown in Table 2. Statistically significant values of *F* and *p* derived from the two-way ANOVA analysis of *CYP* mRNA levels and CYP activity are shown in Tables 1 and 3, respectively.

## Discussion

This is the first report showing changes in the cytochrome P450 expression and function under agomelatine treatment. The obtained results indicate that chronic administration of the novel antidepressant drug agomelatine produces broad changes in the cytochrome P450 expression and activity, which are different from those elicited by the classical tricyclic antidepressant imipramine. Moreover, chronic mild stress (CMS) modifies the effects of antidepressants on the expression of cytochrome P450 genes, but its influence on the enzyme activity is far less pronounced (summarized in Table 4).

**Table 4:** Summary of the effects of CMS and the antidepressant drugs imipramine and agomelatine on the expression and activity of liver cytochrome P450 enzymes

	CYPs	1A1	1A2	2A1	2A2	2B1	2B2	2C6	2C11	2D1		2D2	3A1	3A2
CMS vs nonstressed control	Activity	–		–		–		–	( ↑)		–		–	
	mRNA	–	–	–	–	↓	–	–	↑	–		–	–	–
Imipramine vs nonstressed control	Activity	( ↑)		–		↑		↑	↑	↓			–	
	Protein	–		–		↑		↑	↑	–			↑	↑
	mRNA	( ↑)	( ↑)	–	–	↑	↑	↑	–	–		–	↑	↑
CMS + imipramine vs stressed control	Activity	( ↑)		–		↑		↑	↑	↓			↑	
	Protein	↑		–		↑		↑	↑	–			↑	↑
	mRNA	↓	↓	–	↓	↑	↑	–	↓	–		↓	( ↑)	↑
Agomelatine vs nonstressed control	Activity	↑		↑		( ↑)		↑	↓	↓			↓	
	Protein	↑		↑		↑		↑	↓	–			–	↓
	mRNA	–	↑	↑	↑	↑	↑	↑	↓	–		↑	–	↓
CMS + agomelatine vs stressed control	Activity	↑		↑		( ↑)		↑	↓	–			–	
	Protein	↑		–		–		↑	↓	( ↓)			↓	↓
	mRNA	–	–	–	–	↑	↑	–	↓	–		–	–	↓

↑, ↓ increase or decrease, respectively; (↑), (↓) a tendency to increase or decrease, respectively; − no change

### Imipramine

The reference antidepressant imipramine at the applied dosage (10 mg/kg ip. once a day for 5 weeks) displayed broad-spectrum effects on cytochrome P450 enzyme complex. Imipramine strongly increased the *CYP2B1/2* mRNA levels in nonstressed and stressed rats (in spite of decreased *CYP2B1* mRNA by CMS) and moderately enhanced those of *CYP3A1/2,* which was in line with enhancement of the respective enzyme protein levels (CYP2B, CYP3A1/23 and CYP3A2) in both groups of animals. Accordingly, the activity of CYP2B increased in both groups of animals, and this substantial effect was much more pronounced than that observed previously after a shorter 2-week treatment when only a tendency to increase the CYP2B activity was noticed [42].

However, in spite of the increased expression in both groups, the activity of CYP3A was enhanced by imipramine in stressed rats only, which may be connected with a higher production of imipramine reactive metabolites in nonstressed animals that inactivate the enzyme. The possibility of formation of irreversible CYP-iron(II)-nitrosoalkane complexes by imipramine was demonstrated in vitro [59] and confirmed in vivo [52]. It seems, therefore, that the final effect of imipramine on CYP3A activity depends on a balance between enzyme induction and inhibition.

Similarly, imipramine strongly decreased the activity of CYP2D, not affecting significantly the enzyme expression in both groups of animals, which might be caused by inhibitory action of reactive metabolites, i.e. epoxide intermediates formed during hydroxylation of imipramine, on CYP2D protein activity [60]. The decreased CYP2D activity in rat liver microsomes after in vivo treatment with imipramine was observed already after 1-day exposure to the antidepressant and maintained for 2 weeks [38], which substantiates the above-proposed explanation of the imipramine effect on the enzyme [39].

Imipramine significantly increased the CYP2C6 and CYP2C11 activities and protein levels in both nonstressed and stressed animals. However, the *CYP2C6* mRNA was increased only in nonstressed rats, while the *CYP2C11* mRNA was surprisingly decreased in stressed animals treated with imipramine (in spite of increased the *CYP2C11* mRNA by CMS alone). The above results point to the action of imipramine on CYP2C6 at transcriptional and on CYP2C11 at posttranscriptional level (considering nonstressed rats), and modification of imipramine effects by CMS at transcriptional level. It is theoretically possible that CMS may exert a dual action on cytochrome P450 expression. By increasing corticosterone level, CMS may stimulate CYP gene expression. However, due to its action on the immune system and increased production of pro-inflammatory cytokines, CMS may negatively affect *CYP* gene expression [61, 62]. The final result depends on the intensity of the two mechanisms and their significance for expression of a particular *CYP* gene.

Imipramine decreased both *CYP1A1* and *CYP1A2* mRNA levels in stressed animals, which was not reflected by alterations in the enzyme protein level and activity. It suggests that CMS triggers the inhibitory effect of imipramine on the *CYP1A* gene transcription followed by posttranscriptional modification.

Generally, it may be assumed that the observed effects of chronic treatment with imipramine on cytochrome P450 expression may be partly due to the inhibitory effect of the parent compound and its main metabolite desipramine on noradrenaline uptake which increases brain noradrenergic neurotransmission and thus stimulates the positive central neuroendocrine regulation of enzyme expression [3]. Moreover, an increased noradrenergic transmission decreases proinflammatory cytokines that negatively regulate most of the investigated CYP isoforms [1, 62–64]. On the other hand, the inhibitory effect of the parent drug imipramine on serotonin reuptake leads to the stimulation of brain serotonergic system and negative regulation of the *CYP2C11* expression via neuroendocrine mechanism involving 5-HT_1A_ receptors in the hypothalamic paraventricular nuclei and somatostatin-growth hormone pathway [5, 6, 9]. However, as mentioned elsewhere, the effects of imipramine on cytochrome P450 evoked by the above-described mechanisms may be modified by stress and reactive metabolites of the antidepressant.

### Agomelatine

Agomelatine elicited miscellaneous changes in cytochrome P450 expression and activity, which were similar, different or opposite compared to those produced by imipramine. Similarly to imipramine, agomelatine at the applied optimal dosage (40 mg/kg ip, once-daily for 5 weeks) significantly increased the *CYP2B1* and *CYP2B2* mRNA levels in nonstressed and stressed rats. However, the CYP2B protein level was enhanced only in nonstressed animals, while the enzyme activity tended to increase in both groups. This suggests CMS-induced modification of agomelatine effect at a posttranscriptional or posttranslational level. It was found that an increase in enzyme protein phosphorylation might diminish the CYP2B activity [65] or might predispose the enzyme to degradation [66]. It is conceivable that stress-induced elevation in the concentrations of peripheral catecholamines might affect hepatocyte signaling pathways and the CYP2B protein phosphorylation and, in turn, enzyme protein level and activity in agomelatine-treated rats.

Like imipramine, agomelatine also raised the mRNA and protein level and activity of CYP2C6 in nonstressed rats. This indicates stimulation of *CYP2C6* gene transcription by agomelatine, which was then translated into reinforced protein synthesis and reflected by enhanced enzyme activity in nonstressed rats. This effect of agomelatine was affected by stress at transcriptional level, since no significant change in the *CYP2C6* mRNA level was observed in stressed animals, though the enzyme protein level and activity were slightly increased in the CMS group.

Unlike imipramine, agomelatine reduced the mRNA and enzyme protein level and activity of CYP2C11 and CYP3A2 in both nonstressed and stressed rats and diminished the *CYP3A1/23* mRNA and protein level in stressed animals. The above results indicate negative regulation of *CYP2C11* and *CYP3A2* expression by the antidepressant at a transcriptional level and suggest modification of *CYP3A1/23* expression by CMS in agomelatine-treated rats. The observed negative regulation of the expression of the two *CYP2C11* and *CYP3A2* genes by agomelatine may be caused by the ability of the antidepressant to block 5-HT_2C_ receptors [21–23]. Our recent study has shown that 5-HT_2_ receptors in the arcuate nuclei of the hypothalamus are engaged in the positive neuroendocrine regulation of cytochrome P450 (CYP2C11 and CYP3A) by the stimulation of hypothalamic GHRH secretion and pituitary growth hormone (GH) release and an increase in the serum GH concentration [7, 8]. GH is known to be a positive regulator of the expression of the main rat CYP isoforms CYP2C11 and CYP3A, which are engaged in the metabolism of steroids and drugs.

Surprisingly, the mRNA of *CYP2D1/2* genes (encoding main liver CYP2D enzymes), which are considered as noninducible in the liver, gently rose under agomelatine treatment, which was not reflected by the amount of CYP2D protein, while the enzyme activity was even slightly decreased in nonstressed rats suggesting some drug-induced enzyme inhibition. The latter might possibly be caused by reactive epoxide-metabolites of agomelatine [44]. Interestingly, repeated administration of methamphetamine increased the CYP2D activity, measured using dextromethorphan as a specific substrate in the isolated perfused rat liver model, which suggested enzyme induction [67].

In contrast to imipramine, agomelatine markedly influenced CYP1A and CYP2A enzymes. Agomelatine significantly increased the *CYP1A2* mRNA level in nonstressed rats, but the CYP1A protein level and activity considerably rose in both groups of rats. Agomelatine also raised the mRNA and protein level of *CYP2A1/2* in nonstressed rats, while the CYP2A activity was enhanced in both groups of animals. The above-mentioned observations suggests some modification of the antidepressant effect by CMS at a transcriptional (*CYP1A2* and *CYP2A1/2*), a posttranscriptional (*CYP1A*) or a posttranslational (CYP2A) level. As mentioned before, stress-induced increase in inflammatory cytokines may diminish the expression of *CYP* genes. Moreover, stress-induced changes in phosphorylation processes can affect cytochrome P450 degradation, catalytic activity, substrate binding/specificity and binding of redox partners [66]. Therefore, further molecular investigations are necessary to reveal the mechanism by which CMS affects the CYP1A and CYP2A expression and activity in agomelatine-treated rats.

Thus chronic agomelatine affects all the investigated cytochromes P450 at the level of *CYP* gene regulation, expression or enzyme function (activity). Some of its effects were modified by stress (the mRNA or protein level of CYP1A/2A/2B/3A1) and possibly by reactive metabolites of agomelatine (the CYP2D activity).

In summary, agomelatine and imipramine produced different broad-spectrum effects on cytochrome P450 expression and activity, which suggests the involvement of pharmacological mechanisms and formation of reactive metabolites in the enzyme regulation. CMS modified the effects of antidepressants, which might be caused by alterations in the function of the brain nervous system [61], affecting in this way peripheral glucocorticoids, catecholamines and cytokines and, in turn, hepatic signaling pathways [13, 14, 62, 68]. Although CMS affected antidepressant effects on cytochrome P450 expression, the enzyme activity remained similar. The observed inconsistencies between the levels of mRNA, protein and enzyme activities suggest some posttranscriptional modifications. This may happen, e.g., when reactive drug metabolites are formed (which may interact with RNA or enzyme protein) or when cell signaling is altered (e.g., cAMP production), thereby affecting the phosphorylation processes and, in turn, activation of transcription factors or enzyme protein synthesis, function or degradation [39, 65, 66].

### Conclusions

We conclude that the following: (1) the investigated antidepressants produce different broad-spectrum effects on cytochrome P450 expression and activity: like imipramine, agomelatine increased the expression/activity of CYP2B and CYP2C6, and decreased the CYP2D activity, but unlike imipramine, agomelatine raised the expression/activity of CYP1A, CYP2A, and reduced that of CYP2C11 and CYP3A, which implicates the possibility of drug–drug interactions during combined treatment; (2) the different effects of antidepressants suggest the involvement of central and peripheral regulatory mechanisms as well as interactions between the enzyme and drug reactive metabolites; (3) CMS influences the effects of antidepressants on cytochrome P450 expression (mRNA, enzyme protein), but does not change appreciably their effects on the enzyme activity. This suggests that the rate of antidepressant drug metabolism under CMS is similar to that under normal conditions.

Further molecular studies need to be carried out to elucidate the mechanisms underlying antidepressant/CMS action on cytochrome P450 expression and activity. Moreover, plasma concentrations of agomelatine after the applied dose should be measured in rats under CMS and compared to those observed in patients upon antidepressant treatment to translate the obtained results on humans.
